# Neuronal specializations for the processing of interaural difference cues in the chick

**DOI:** 10.3389/fncir.2014.00047

**Published:** 2014-05-09

**Authors:** Harunori Ohmori

**Affiliations:** Department of Physiology and Neurobiology, Faculty of Medicine, Kyoto UniversityKyoto, Japan

**Keywords:** brainstem auditory nucleus, interaural difference cues, SON, tonic inhibition, phasic inhibition

## Abstract

Sound information is encoded as a series of spikes of the auditory nerve fibers (ANFs), and then transmitted to the brainstem auditory nuclei. Features such as timing and level are extracted from ANFs activity and further processed as the interaural time difference (ITD) and the interaural level difference (ILD), respectively. These two interaural difference cues are used for sound source localization by behaving animals. Both cues depend on the head size of animals and are extremely small, requiring specialized neural properties in order to process these cues with precision. Moreover, the sound level and timing cues are not processed independently from one another. Neurons in the nucleus angularis (NA) are specialized for coding sound level information in birds and the ILD is processed in the posterior part of the dorsal lateral lemniscus nucleus (LLDp). Processing of ILD is affected by the phase difference of binaural sound. Temporal features of sound are encoded in the pathway starting in nucleus magnocellularis (NM), and ITD is processed in the nucleus laminaris (NL). In this pathway a variety of specializations are found in synapse morphology, neuronal excitability, distribution of ion channels and receptors along the tonotopic axis, which reduces spike timing fluctuation in the ANFs-NM synapse, and imparts precise and stable ITD processing to the NL. Moreover, the contrast of ITD processing in NL is enhanced over a wide range of sound level through the activity of GABAergic inhibitory systems from both the superior olivary nucleus (SON) and local inhibitory neurons that follow monosynaptic to NM activity.

## Introduction

**Figure 1:**
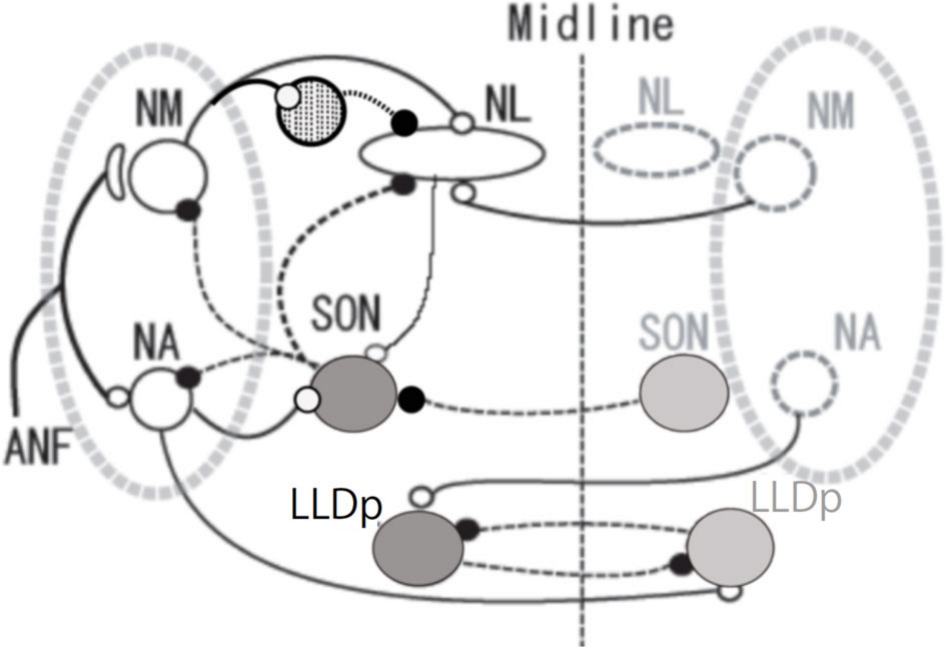
**Schema of avian auditory brainstem nuclei**. Symbols indicate as follows: large open circles (excitatory neurons), large gray circles (inhibitory neurons), small open circles and solid lines (excitatory inputs) small filled circles and dashed lines (inhibitory inputs), and circle filled with dots (local inhibitory neurons). The local inhibitory neurons receive monosynaptic excitatory input from the ipsilateral NM. ANF, auditory nerve fiber; NM, nucleus magnocellularis; NA, nucleus angularis; NL, nucleus laminaris; SON, superior olivary nucleus; LLDp, posterior part of dorsal lateral lemniscus nucleus.

The auditory nervous system is highly sensitive to changes in acoustic signals both in the frequency and the level (Dooling et al., 2000; Klump, 2000). Activity of ANFs codes the sound timing as the phase-locked-firing and the level as the firing-rate. Anatomically separate, and physiologically distinct pathways process these two auditory features (Oertel, 1999; Carr and Code, 2000). Anatomical separation is particularly distinct in the avian auditory system (Figure 1), where the pathway starting from NM carries the temporal information, and ITD is processed in NL. The pathway starting from NA carries the intensity information and ILD is processed in LLDp (Sullivan and Konishi, 1984; Takahashi et al., 1984). These two interaural differences inherent in auditory signals are used as cues for sound source localization (Moiseff, 1989). ITDs are generally used for processing low frequency sounds, while ILD is a cue used for high frequencies (Rayleigh, 1907).

Sharpening of ITD selectivity by GABAergic inputs has been demonstrated in higher auditory nuclei such as the inferior colliculus of the barn owl (Fujita and Konishi, 1991). In mammals, neurons in medial superior olive receive glycinergic inhibitory innervation from the medial and the lateral nucleus of the trapezoid body (Kuwabara and Zook, 1992; Grothe and Sanes, 1994). We are therefore interested in the presence and the roles of such inhibitory innervations in the ITD processing of NL. GABAergic innervations in NL are mostly from neurons in SON and some from the GABA positive interneurons located near NM and NL (Code et al., 1989; von Bartheld et al., 1989; Yamada et al., 2013). SON receives excitatory inputs from ipsilateral NA and NL, and inhibitory inputs from the contralateral SON, and makes projections to the ipsilateral NL, NM, NA and to the contralateral SON (Figure 1; Lachica et al., 1994; Yang et al., 1999; Monsivais et al., 2000; Burger et al., 2005).

In this review article I will first discuss the possible interplay between ILD and ITD, then I will detail the specializations found in the timing processing pathway, and the role of inhibition to make the ITD tuning tolerant to the sound level.

## ILD processing is affected by interaural phase difference

Timing and level information is processed in separate neuronal pathways originating in the cochlear nuclei but ultimately merge in the midbrain, mesencephalicus lateralis dorsalis (avian homolog of the inferior colliculus; Pena and Konishi, 2001; Konishi, 2003). However, they are not processed in total separation even at lower levels. They influence one another at multiple steps of encoding and processing. Sound level affects processing of ITD under certain conditions (Viete et al., 1997; Dasika et al., 2005; Nishino et al., 2008), and sound timing affects processing of ILD (Sato et al., 2010; in mammals see Finlayson and Caspary, 1991; Joris and Yin, 1995; Tollin and Yin, 2005). ILD is processed in the avian LLDp. LLDp neurons are excited by contralateral sound and inhibited by ipsilateral sound, reflecting excitation by the contralateral NA and inhibition from the ipsilateral NA through the contralateral LLDp as it is detailed in the barn owl (Figure 1; Manley et al., 1988; Mogdans and Knudsen, 1994).

**Figure 2:**
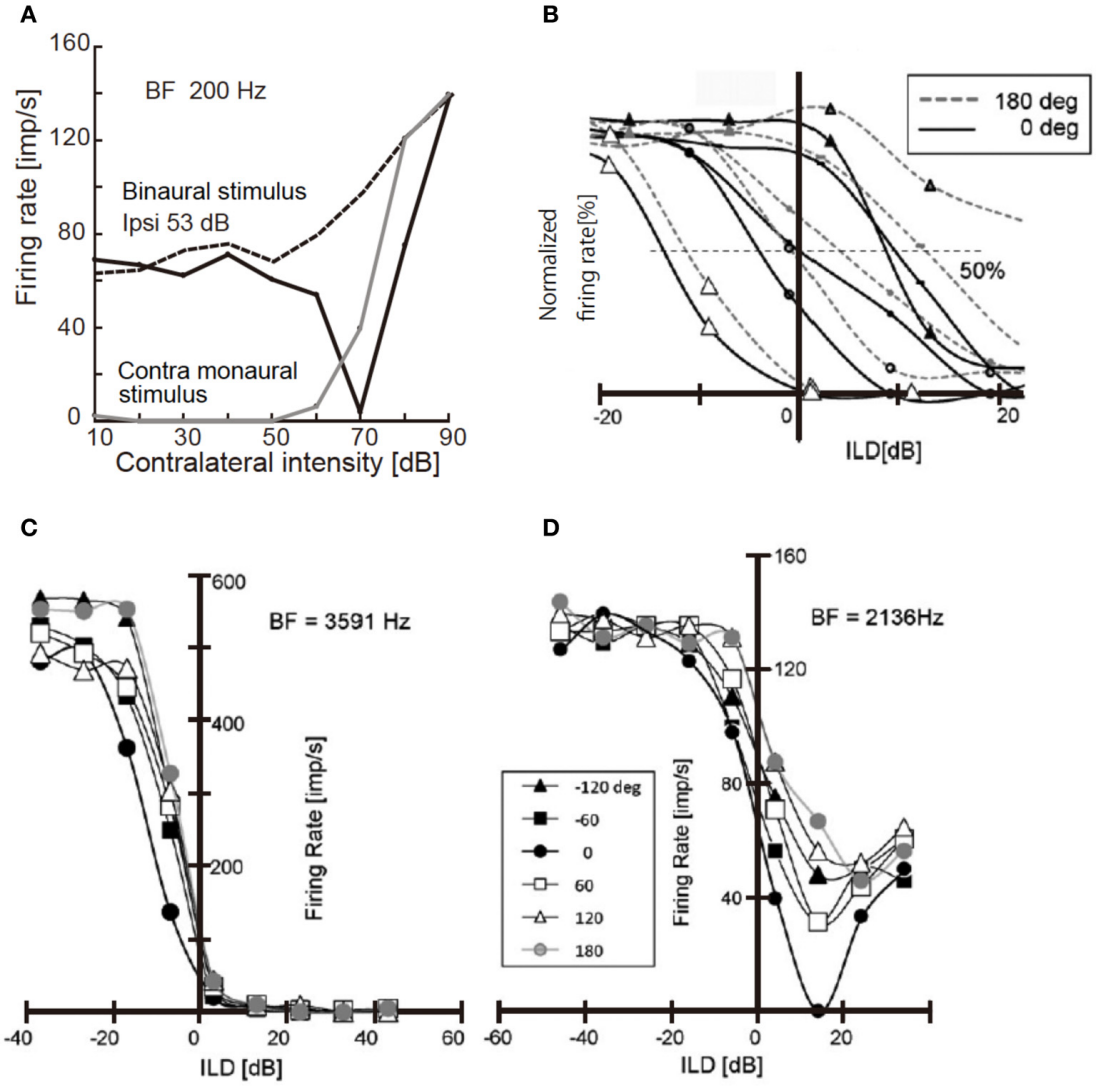
**Responses of NA unit and LLDp units to binaural stimuli**. **(A)** Response of NA units to contralateral tone of 200 Hz. Binaural stimulus was applied in-phase (solid black line) or 180° out of phase (dotted line) with a constant ipsilateral tone of 53 dB; 20 dB above the threshold. Responses induced by contralateral monaural stimulus are included (solid gray line). **(B)** Rate-ILD relationship of LLDp units. ILD was defined as ipsi-contra SPL [dB]. Different symbols indicate different cells. Solid lines are for in-phase and broken lines are for 180° out-of-phase relationships. **(C,D)** IPD modulation of rate-ILD relationship of LLDp units with strong ipsilateral inhibition **(C)** with weak ipsilateral inhibition **(D)**. The inset indicates IPDs applied to both **(C,D)**. The slope of rate-ILD relationship across zero ILD is affected by IPD in **(C)** but not in **(D)**. Reproduced with permission from Sato et al. (2010).

Neural activity in NA and LLDp is changed with sound location, and is affected by the interaural phase difference (IPD). IPD modulates the activity of NA neurons in the chick through the acoustic interaction across the interaural canal that connects the middle ear cavities of two sides (Hyson et al., 1994; see also Christensen-Dalsgaard et al., 2011). The activity of NA neurons is suppressed by strong contralateral tones when binaural stimuli were presented in-phase, but activity increased monotonically with sound level when dichotic tones were at 180° out-of-phase (Figure 2A; Sato et al., 2010). Consequently, IPD dependence of firing activity of the NA neuron affects the ILD processing of the LLDp units of the chick (Figure 2B). However, in the barn owl, because of a sharp attenuation of the acoustic coupling across the interaural canal at frequencies above 3 kHz, the acoustic binaural interaction is negligible (Moiseff and Konishi, 1981).

The firing rate of LLDp units increases with increasing contralateral sound level, and decreases with increasing ipsilateral sound level. Moreover, the strength of inhibition by ipsilateral sound level varied among LLDp units, and a group of LLDp neurons was inhibited strongly by the ipsilateral sound (Figures 2B,C). IPD affected the rate-ILD function of LLDp neurons, and LLDp neurons that were inhibited strongly enhanced the selectivity toward the contralateral ear through the modulation of rate-ILD function (Figures 2C,D). The ratio of slopes of rate-ILD relationship between the contralateral dominant sound and the ipsilateral dominant sound across 0 ILD indicates the direction selectivity of LLDp units. This IPD effect on ILD processing in LLDp neurons may compensate for the small ILD cue available to the animal (Sato et al., 2010). The balance of excitation and inhibition changes with sound location, and in the barn owl LLDp, it is reported that the reliability of the response to spectrotemporal feature of LLDp neuron is enhanced by temporally delayed inhibition of LLDp neurons through gain modulation of the input-output function of the neuron (Steinberg et al., 2013).

## Synaptic specializations in NM

Neurons in NM do not have appreciable dendrites, and ANFs make synapses on the cell soma. ANFs form enfolding end-bulbs of Held around the cell body in the high and middle characteristic frequency (CF) neuron but not in low CF neurons. Accordingly the EPSCs recorded in the high-middle CF NM neurons are large and generated in all-or-none manner with a small number of amplitude steps when the intensity of electrical stimulation applied to the ANFs bundle is changed, while the EPSCs recorded in the low CF neurons are small and the size gradually increases depended on the intensity of electrical stimulus (Fukui and Ohmori, 2004). NM neurons express low-voltage-activated Kv1.1 channels with a gradient along the tonotopic axis. High CF neurons have stronger Kv1.1 channel expression and conductance, resulting in more negative resting membrane potential and higher spike threshold. Blocking these channels by dendrotoxin depolarizes the resting membrane potential and reduces the spike threshold (Fukui and Ohmori, 2004). Dendrotoxin is known to block low-voltage-activated K^+^ channels of Kv1.1, Kv1.2, and Kv1.6 subtypes (Hopkins et al., 1994; Harvey, 2001). Synaptic transmission during on-going stimuli is robust in the high-middle CF synapse but is depressed quickly in low CF synapses (Oline and Burger, 2014). A large readily releasable pool size in the high-middle CF terminals could maintain the reliable transmission. This may function to maintain the suprathreshold EPSCs in high CF neurons while enabling summation to enhance phase-locking in low CF neurons as it is discussed below.

NM neurons are specialized to encode temporal information of sound from ANFs activity. The low frequency sound information is strongly phase-locked, however it is actually encoded with a large timing jitter in ANFs. This timing fluctuation is reduced during transmission from ANFs to NM neurons (Fukui and Ohmori, 2004; Fukui et al., 2006; see Joris et al., 1994). Here, the mechanism is explained by the temporal integration of small EPSPs. Because the low frequency NM neuron is innervated by a large number of small bouton shaped synapses, single EPSPs are so small that multiple EPSPs are required to summate in order to reach spike threshold (Fukui and Ohmori, 2004; Kuba and Ohmori, 2009). Therefore, only those synaptic inputs arriving within a limited time window could contribute to NM spike; NM activity becomes more precisely phase-locked than ANF activity. However, the integration makes the depolarization of the NM neuron slow, which increases the level of inactivation of Na^+^ channels. Axon initial segment (AIS), the site of action potential initiation, is extended longer in the axon of low CF NM neurons than the high-middle CF NM neurons. Clustering of a large number of Na^+^ channels at the AIS would allow sufficient current to generate action potentials even under a certain level of inactivation (Kuba and Ohmori, 2009). On the other hand, high frequency neurons are innervated by a small number of end-bulb shaped large terminal of ANF. Large EPSCs are generated and timing information is transmitted more precisely to high frequency NM neurons (Fukui and Ohmori, 2004; Fukui et al., 2006; Oline and Burger, 2014).

## Specializations of ITD encoding in the NL

Somas of NL neurons have bipolar tufted dendrites and an axon emerges from the cell body. Dentrite morphology changes systematically along the tonotopic axis. Dendrites are short, relatively unbranched, occur in large numbers in high CF NL cells. The number of dendrites decreases in the middle-CF neurons but they become thicker and longer. Only a few primary dendrites extend away from the soma in the low-CF neurons, and they have extensive branching (Smith and Rubel, 1979; Kuba et al., 2005; Sanchez et al., 2010).

ITD depends on head size, and in most birds, the physiological maximum ITD is smaller than 100 μs. Considering the maximum firing rate of most neurons is less than or equal to 1 kHz, this maximum available ITD cue is extremely small; thus the auditory system needs specialization to process ITDs accurately.

During embryonic development, NMDA receptor currents increase in the NM-NL synapse, however it decreases dramatically before hatching. AMPA receptor currents increase during the embryonic development, particularly in the high CF NL cells. The EPSC kinetics becomes faster with development and rectifies in all CF regions, suggesting the exclusion of GluR2 receptor subunits from the synapse (Sanchez et al., 2010). Kinetics and amplitude of EPSCs are symmetrical in single NL neurons between inputs of two sides (Lu, 2009). Moreover, tonotopic gradients are matched between the EPSC time course and the feature of postsynaptic band-pass filtering in single NL neurons (Slee et al., 2010). These are consistent with the faster EPSC and mEPSC kinetics in NL neurons after hatching (Kuba et al., 2005).

### Low-voltage-activated K^+^ channels enhance coincidence detection, and make ITD detection most sensitive for mid-frequency sound

**Figure 3:**
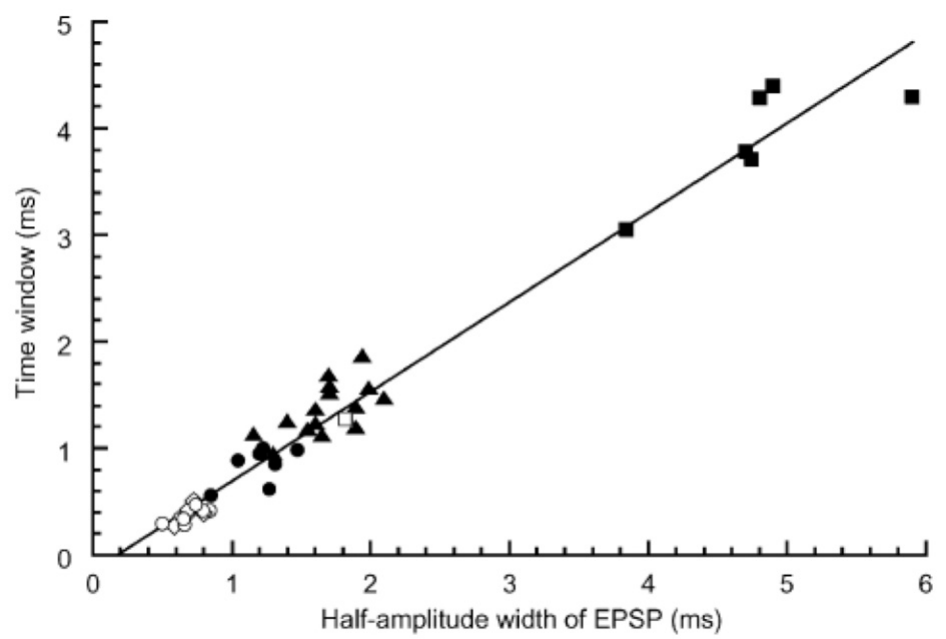
**Correlation between the time window for coincidence detection and the width of EPSP measured in the NL neurons**. Time windows were plotted against the half amplitude width of EPSP recorded at −62 mV. Different symbols indicate different experimental conditions: ▪, embryo recorded at 20–25°C (*n* = 6); □, embryo 40°C (*n* = 1); ▴, chick 20–25°C (*n* = 15); ●, chick 30°C (*n* = 7); ○, chick 40°C (*n* = 8); ◊, chick 40°C without bicuculline using low-Cl internal solution (*n* = 5). Reproduced with permission from Kuba et al. (2003).

The best sensitivity to ITD or the smallest error of sound source localization was observed in the mid-audible frequency range in the avian species (Klump, 2000). Consistent with this observation, we found that the coincidence detection of bilateral NM spikes was most accurate in the middle-CF NL neurons. In brainstem slice experiments of the post-hatch chicks conducted at body temperature, 40°C, the time window of coincidence detection was 1700, 300, and 600 μs, for the low, middle and high CF neurons, respectively; the time window is defined as the time separation of bilateral stimuli applied to projection fibers from NM, which generates spikes in more than 50% trials (Kuba et al., 2003). Moreover, we found that the time course of EPSP measured as the half amplitude width have a significant positive correlation with the time window of coincidence detection (Figure 3); therefore, NL neuron with fast EPSPs has temporally sharp coincidence detection. The time course of EPSC is progressively faster toward high CF neurons. However, the time course of EPSP is fastest in mid-CF neurons, which is almost the same or sometimes faster than the time course of EPSC recorded in the same neuron (Kuba et al., 2005). The falling phase of the EPSP was accelerated due to strong activation of low-voltage-activated K^+^ channels caused by EPSPs. Application of dendrotoxin prolonged the falling phase of EPSP. The expression of Kv1.2 channels is confirmed immuno-histochemically in the NL, and the density of immuno-reactivity is the highest in the mid-CF region, where the time window for the coincidence detection is most precise (Kuba et al., 2005). These findings are consistent with the idea that Kv1.2 channels accelerate EPSP time course in the middle-CF NL neurons.

### Na^+^ channel distribution in AIS makes spike-generation stable in wide frequency ranges

We have been puzzled for a long time by the observation that the spikes and Na^+^ currents were small in the high and middle CF NL neurons than those of low CF NL neurons (Kuba et al., 2003, 2005, 2006). By immuno-histochemical observations we found that the AIS is extended in length and located close to the cell soma in low CF NL neurons while short and located distant in the high CF NL neurons. The significance of this Na^+^ channel distribution is interpreted by a computer simulation using a NEURON model under an assumption that NL neurons receive excitatory synaptic inputs at the frequency that closely matches with their CF; namely the frequency of synaptic inputs is high in the high CF NL neurons and low in the low CF NL neurons. Simulations demonstrated that the depolarization of the cell soma is greater in high CF NL neurons than the low CF NL neurons during sound inputs. This depolarization would inactivate Na^+^ channels and prevent spike generation if the AIS, thus Na^+^ channel, is located close to the cell soma. By displacing the AIS to a distance where the level of steady depolarization is small because of the electro-tonic property of the axon, the level of Na^+^ channel inactivation should be reduced; however the reduced level of membrane depolarization may also reduce the activation level of Na^+^ channels at a distance. Consequently, the balance of activation and inactivation of Na^+^ channels is achieved, and the spike generation is optimized by controlling the spatial distribution of Na^+^ channels for each NL neuron depending on its CF. This is likely the underlying mechanism for the stable processing of ITD in each NL neuron (Kuba et al., 2006; see also Ashida et al., 2007).

### HCN channels modify the coincidence detection

Hyperpolarization-activated cyclic nucleotide-gated (HCN) channels have a reversal potential around −30 mV, are activated by membrane hyperpolarization, and the voltage-sensitivity is modulated by cyclic nucleotides. Channel gating is shifted in the positive direction when the cytosolic concentration of cyclic nucleotides is high, and the sensitivity to cyclic nucleotide is greater in HCN2 than in HCN1 channel subtype (Pape, 1996; Santoro and Tibbs, 1999; Biel et al., 2009). In the chicken NL, both HCN1 and HCN2 channels are expressed along the tonotopic axis with a gradient (Yamada et al., 2005). Expression of HCN1 is graded extensively toward the low CF region of the nucleus, while the expression of HCN2 is less graded across the nucleus. The membrane depolarization of NL neurons was confirmed when the level of cyclic AMP was raised either by incubation of slices with 8-Br-cAMP or by photo-illumination of the cell that was loaded with a caged compound of cyclic AMP through the patch electrode, which likely reflected an increased level of activation of HCN channels (Yamada et al., 2005). The membrane depolarization improved the coincidence detection by accelerating the time course of EPSPs, presumably because of the activation of low-voltage-activated K^+^ channels. The relatively high density of HCN2 channels over HCN1 channels in the high CF NL neurons made the high CF neurons more sensitive to the level of cyclic AMP (Yamada et al., 2005). Accordingly, by incubation of slices prepared from the high CF NL region with nor-adrenaline for a few minutes, the coincidence detection became more precise. Nor-adrenaline is a neurotransmitter released from sympathetic nerve terminals and is expected to activate G-protein-coupled receptors and increase cyclic AMP concentration in the target neurons (Gilman, 1987). These results raise the possibility that coincidence detection is under sympathetic control. HCN channel activity could be coupled with the improved sound source localization capability of the barn owl observed when owls were exposed to a sound stimulus of long duration (Knudsen and Konishi, 1979). Listening to a sound of long duration may increase the tension that likely mobilizes the sympathetic activity. Expression pattern of HCN channel subunits in the owl has not yet been examined.

### Metabotropic glutamate receptors (mGluRs) enhance the low frequency coincidence detection

The fast time course of EPSPs is critical to enhance the coincidence detection; however the sharpness of coincidence detection depends also on the size of EPSPs (Kuba et al., 2002). The size was not only affected by the short term synaptic plasticity, but was affected through the presynaptic mGluR activity as well (Okuda et al., 2013). A non-specific agonist of mGluRs (t-ACPD) reduced the amplitude of EPSCs, which reduced the depression of EPSCs during a stimulus train, while the paired pulse ratio and the coefficient of variation of EPSC amplitude were increased. In contrast, the amplitude of spontaneous EPSCs was not affected, but the frequency was reduced. Thus, the effects of t-ACPD were presynaptic and t-ACPD likely reduced the release of neurotransmitter from the NM terminal. Both group-II (DCG-IV) and group-III (L-AP4) specific agonists reduced EPSC amplitude by presynaptic mechanisms, and the effects were greater in low CF NL neurons. The reduced EPSP amplitude in DCG-IV improved the coincidence detection. A specific antagonist of group-II mGluRs (LY341495) increased the amplitude of both EPSCs and EPSPs, and enhanced depression during the stimulus train, which indicated a constitutive activation of mGluRs in the NL even though experiments were conducted in slice preparations. We have detected expression of group-II mGluRs immuno-histochemically, and the expression level was increased after hatching. The expression was greater toward the low CF NL region. These observations indicate that the presynaptic mGluRs may operate as a self-regulatory mechanism to optimize the size of EPSP and have roles in sharpening the coincidence detection, particularly during the on-going sound stimulus.

## Inhibitory synapses in the NL

Because of the relatively high intracellular concentration of Cl^−^, GABA was depolarizing in brainstem auditory neurons (Hyson et al., 1995). GABA-induced depolarization could exceed the spike threshold and could be excitatory; however GABA application reduced input impedance and was primarily inhibitory. Therefore, sustained GABA effects are critical in improving the temporal processing of sounds (Funabiki et al., 1998; Tang et al., 2011). Moreover, GABAergic inhibitory synapse was affected by GABA_B_ receptors and mGluRs in NL of embryonic age (E19-E21, Tang et al., 2009). These GABA_B_ and mGluRs are cooperative and may improve the coincidence detection in NL neurons.

### Sustained gabaergic inhibition improves ITD processing

Firing rates of ITD processing neurons alternates periodically as ITD changes during a tonal stimulation, and the period of the ITD tuning curve was determined by the CF of the neuron (Goldberg and Brown, 1969; Carr and Konishi, 1990; Yin and Chan, 1990). The sound pressure level affected the contrast between the peak and trough firing rates (Pena et al., 1996). Loud sound was expected to increase the firing rate both at the peak and the trough of ITD tuning curve, and to reduce the peak-trough contrast (or ITD sensitivity, Dasika et al., 2005). However, the peak-trough contrast was actually maintained rather than reduced at high sound pressure level in *in vivo* recordings from the barn owl (Pena et al., 1996). Pena and colleagues proposed that inhibition from SON controls the ITD tuning in NL, making it tolerant to sound pressure level.

By recording single unit activity in NL *in vivo*, ITD tuning was found dependent both on the sound frequency and the sound pressure level (Nishino et al., 2008). The peak-trough contrast in mid-to-high CF NL units (higher than 1 kHz) was maximal at intermediate sound pressure levels. The peak-trough contrast was practically lost when a very loud sound was applied because of the increased firing rate both at the peak and the trough of ITD tuning curve (90 dB or louder sound). In low CF NL units (lower than 1 kHz), neural activity was temporally suppressed after a loud sound. The peak-trough contrast became larger as the sound became louder. This is because the trough-firing rate decreased with the sound pressure level, even to the level lower than the spontaneous firing rate. These observations are consistent with the sustained SON inhibition of low CF NL neurons. Consistently after electrical lesioning of the ipsilateral SON the contrast of ITD tuning in the low CF NL neuron collapsed at loud sound (Nishino et al., 2008), and the tolerance of ITD tuning to the sound pressure level became similar to that of the mid-to-high CF NL units. The sound pressure level dependence of ITD processing of the mid-to-high CF NL neurons was not virtually affected by lesioning of the SON. SON receives sound pressure level information through NA (Figure 1), and GABAergic projection from SON to NL is robust in the low CF region of NL but becomes less prominent toward the high CF region. The density of the SON projection along the tonotopy is correlated with the magnitude of the response to SON lesions across the tonotopic axis in NL (Nishino et al., 2008). We conclude, accordingly, that the dense inhibitory projection from SON to NL makes the ITD tuning tolerant to the sound pressure level in NL (Nishino et al., 2008).

### Phasic inhibition by local gabaergic neurons improves ITD processing when the excitatory input level is low

**Figure 4:**
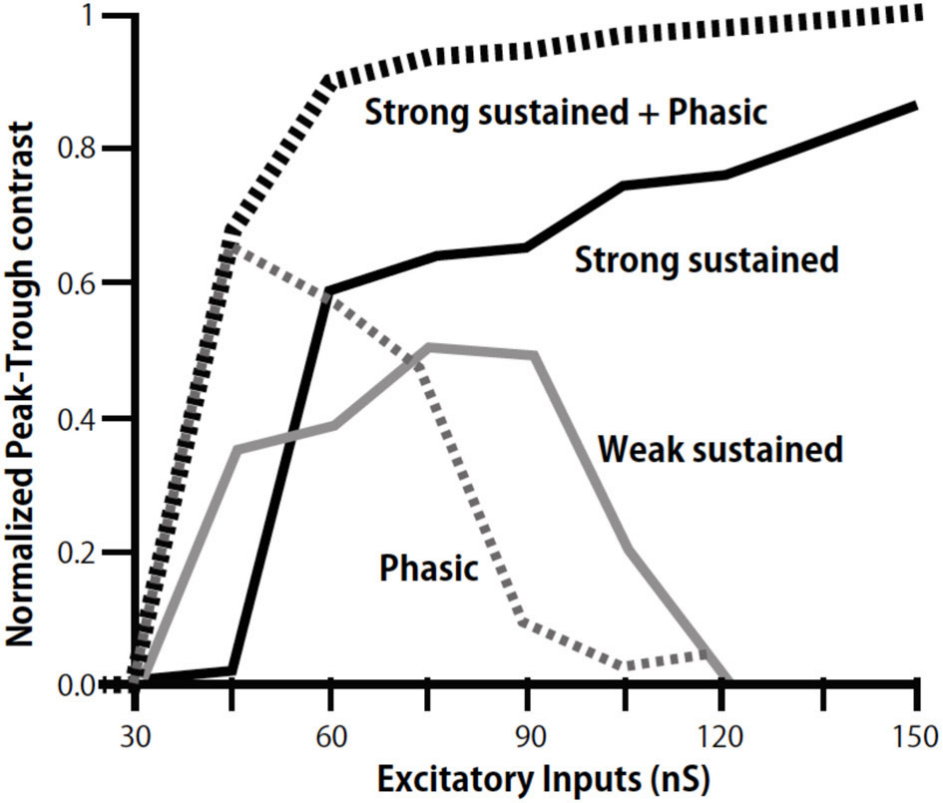
**Modulation of peak-trough contrast of ITD tuning curve of low-CF NL neurons by inhibition**. Peak-trough contrasts of ITD tuning curve are calculated by including the sustained inhibition of weak (gray line), strong (black line), and phasic inhibition (dotted gray line) separately, and by including both the strong sustained and the phasic inhibition (dotted black line). Modified from Yamada et al. (2013).

We further found a phasic IPSC in the low CF NL neurons in slice preparations, which followed the ipsilateral NM inputs with a short time delay and small timing jitter; thus the phasic IPSC likely follows monosynaptically the ipsilateral NM activity (Yamada et al., 2013). GABA-positive small neurons are distributed in and near the NL (Carr et al., 1989; von Bartheld et al., 1989). When photoactivated by a caged glutamate compound these neurons generated IPSC in NL neurons suggesting that these GABAergic neurons are interneurons that mediate the phasic inhibition. These IPSCs in the low CF NL region have fast decay kinetics that is attributable to α1 subunit of the GABA_A_ receptor (Goldstein et al., 2002; Eyre et al., 2012), the expression of which is dominated in the low-CF region of the NL. The fast decay kinetics is consistent with the faster kinetics of GABAergic IPSC in the low CF NL neuron observed by Tang and Lu (2012). Simulations using a NEURON model demonstrated that phasic IPSCs increase the contrast of ITD-tuning when the sound pressure level is low. Furthermore, the simulation demonstrated that the cooperation of phasic and sustained inhibitions effectively increases the contrast of ITD-tuning over a wide range of excitatory input levels (Figure 4; Yamada et al., 2013).

## Conclusions

Interaural difference cues are small, particularly for animals endowed with small heads. This review has focused on works conducted on the chick, which provide profound insights into the mechanisms that contribute to the accuracy of ITD processing. Further, these studies reveal how ITD tuning is maintained over a wide range of sound pressure level in birds. The morphological specializations complement the roles of ionic channels in the ITD tuning. The distribution of ionic channels and receptors including the inhibitory synapses in the NL is critically arranged to optimize the ITD processing, and in turn, sound source localization. Moreover, timing and level cues of sounds are used cooperatively in both mammals and birds to improve the processing of small interaural difference cues.

### Conflict of interest statement

The author declares that the research was conducted in the absence of any commercial or financial relationships that could be construed as a potential conflict of interest.
